# Role of ^18^F-FDG PET/CT Radiomics Features in the Differential Diagnosis of Solitary Pulmonary Nodules: Diagnostic Accuracy and Comparison between Two Different PET/CT Scanners

**DOI:** 10.3390/jcm10215064

**Published:** 2021-10-29

**Authors:** Domenico Albano, Roberto Gatta, Matteo Marini, Carlo Rodella, Luca Camoni, Francesco Dondi, Raffaele Giubbini, Francesco Bertagna

**Affiliations:** 1 Nuclear Medicine, University of Brescia and ASST Spedali Civili Brescia, 25123 Brescia, Italy; camoni.luca@gmail.com (L.C.); f.dondi@outlook.com (F.D.); raffaele.giubbini@unibs.it (R.G.); francesco.bertagna@unibs.it (F.B.); 2 Dipartimento di Scienze Cliniche e Sperimentali dell’Università degli Studi di Brescia, 25128 Brescia, Italy; roberto.gatta@unibs.it; 3 University of Brescia, 25123 Brescia, Italy; m.marini032@studenti.unibs.it; 4 Health Physics Department, ASST-Spedali Civili, 25123 Brescia, Italy; carlo.rodella@asst-spedalicivili.it

**Keywords:** ^18^F-FDG PET/CT, radiomics, texture analysis, solitary pulmonary nodule, lung cancer

## Abstract

The aim of this retrospective study was to investigate the ability of 18 fluorine-fluorodeoxyglucose positron emission tomography/CT (^18^F-FDG-PET/CT) metrics and radiomics features (RFs) in predicting the final diagnosis of solitary pulmonary nodules (SPN). We retrospectively recruited 202 patients who underwent a ^18^F-FDG-PET/CT before any treatment in two PET scanners. After volumetric segmentation of each lung nodule, 8 PET metrics and 42 RFs were extracted. All the features were tested for significant differences between the two PET scanners. The performances of all features in predicting the nature of SPN were analyzed by testing three classes of final logistic regression predictive models: two were built/trained through exploiting the separate data from the two scanners, and the other joined the data together. One hundred and twenty-seven patients had a final diagnosis of malignancy, while 64 were of a benign nature. Comparing the two PET scanners, we found that all metabolic features and most of RFs were significantly different, despite the cross correlation being quite similar. For scanner 1, a combination between grey level co-occurrence matrix (GLCM), histogram, and grey-level zone length matrix (GLZLM) related features presented the best performances to predict the diagnosis; for scanner 2, it was GLCM and histogram-related features and metabolic tumour volume (MTV); and for scanner 1 + 2, it was histogram features, standardized uptake value (SUV) metrics, and MTV. RFs had a significant role in predicting the diagnosis of SPN, but their accuracies were directly related to the scanner.

## 1. Introduction

A solitary pulmonary nodule (SPN) is defined as a lung lesion smaller than 3 cm in diameter that is completely surrounded by pulmonary parenchyma, without other abnormalities (such as atelectasis or adenopathy) [1,2]. The prevalence of SPN detected by chest X-rays and computed tomography (CT) is wide (range 2–50%) and is increasing [3,4]. The definition of SPN nature may be a diagnostic challenge due to the difficulties of having solid factors that may differentiate malignant lesions from benign lesions. In fact, the potential differential diagnosis of SPN includes malignant diseases, such as primary lung cancer, distant metastases, or rarer lymphoma, as well as benign causes, such as tuberculosis, pneumonia, fungi infections, and primary benign tumors (hamartoma, angioma, etc.) [5,6]. For the discrimination of the nature of SPN, clinical (age, smoke, and exposure to carcinogenic agents) and morphological (size, density, growth, margins, wall thickness, and the presence of cavitation and calcifications) features were investigated, with controversial results [7]. The management of SPN is related to the risk assessed, usually involving routine CT follow-ups, functional imaging with 18 fluorine-fluorodeoxyglucose positron emission tomography/CT (^18^F-FDG PET/CT), and/or tissue sampling. ^18^F-FDG PET/CT showed high sensitivity but moderate specificity [8,9], meaning that false positive results may occur relatively frequently. Semiquantitative PET/CT factors, especially maximum standardized uptake value (SUVmax), were tested, and different SUVmax thresholds were proposed with variable accuracy. Recently, the application of different tools for the extraction of quantitative imaging features (called radiomics) has become of particular interest as a possible way to discriminate between malignant and benign lesions [10], and also in the study of SPN some preliminary pieces of evidence are available. Some studies have investigated a range of combinations of radiomics features from CT, proposing predictive models with optimal diagnostic performance (overall accuracy between 70% and 95%) [11,12,13,14,15,16,17,18]. Moreover, PET/CT texture features were studied with promising results [19,20,21,22,23,24]; however, many open issues stay, such as the real meaning of these RFs, the right methodology to follow to calculate RFs and the potential impact of technological features in their measurements. The rationale behind radiomics application is to leverage on that fraction of image information that may have clinical relevance but go unnoticed to the human eye [25]; however, the potential usefulness of this tool is yet unexplored.

Another point not perfectly understood is the potential influence of technology available in the measurements of RFs, such as the type of scanner [26,27].

Thus, the aim of this retrospective study was to analyze whether the texture features from PET/CT could lead to a better discrimination between malignant and benign SPN compared to conventional PET/CT semiquantitative features.

The second point was to investigate the impact of different PET scanners in the measurements of these texture features and how these differences can affect the development of predictive models.

## 2. Materials and Methods

### 2.1. Patients

**Figure 1:**
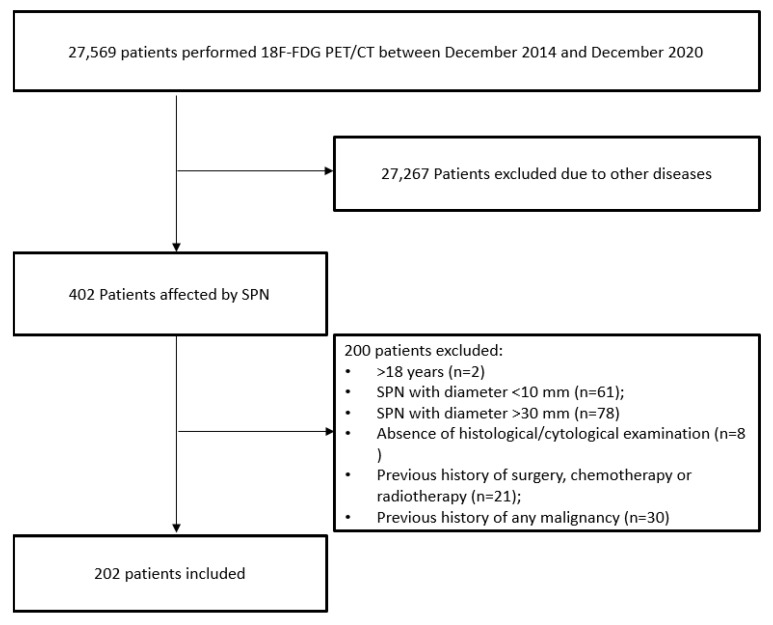
Flowchart of patients included. SPN: solitary pulmonary nodule.

Between December 2014 and December 2020, we retrospectively included 202 patients who underwent a ^18^F-FDG PET/CT scan for the metabolic evaluation of a solitary lung nodule. Inclusion criteria were: (1) >18 years old; (2) the presence of a single solid pulmonary nodule at CT with maximum axial diameter more than 10 mm and up to 30 mm; (3) citologically or histologically confirmation of the final diagnosis of the lung nodule; (4) no previous history of any malignancy; (5) no previous history of surgery, chemotherapy, and/or radiotherapy (Figure 1).

All patients gave written informed consent as part of the PET/CT routine, and their data were treated according to the local privacy rules and laws. Request for an ethical standard was waived due to the retrospective nature of the work.

### 2.2. ^18^F-FDG PET/CT Imaging and Interpretation

All patients underwent baseline ^18^F-FDG PET/CT before any treatment to study SNP detected by a previous radiological examination (chest CT or X-rays). ^18^F-FDG-PET/CT scan was performed after at least 6 h fasting and with glucose level lower than 150 mg/dL. An activity of 3.5–4.5 MBq/kg of ^18^F-FDG was administered intravenously, and images were acquired 60 ± 10 min after injection from the skull basis to the mid-thigh on two PET/CT scanners: a Discovery 690 PET/CT scanner (scanner 1) and a Discovery STE PET/CT scanner (scanner 2) (General Electric Company—Milwaukee, WI, USA) with standard parameters (CT: 80 mA, 120 Kv without contrast; 2.5–4 min per bed-PET-step, axial width 15 cm); the reconstruction was performed in a 256 × 256 matrix and 60 cm field of view. DST PET is characterized by BGO (bismuth germanate crystal) scintillator crystal with a decay time of 300 ns and D690 by LYSO (cerium-doped lutetium yttrium oxyorthosilicate) scintillator crystal with a decay time of 45 ns. The two scanners were not harmonized with a cross calibration program. PET/CT were acquired at free breath, only by instructing the patient to take regular breaths.

For both tomographs a standard non-contrast free-breathing helical low dose CT was obtained for morphologic correlation and attenuation correction. The D-STE acquisition parameters were: 120 kV, fixed tube current ≈73 mAs (40–160 mAs), 4 slices × 3.75 mm and 3.27 mm interval, pitch 1.5:1, tube rotation 0.8 s. The D690 acquisition parameters were: 120 kV, fixed tube current ≈60 mAs (40–100 mAs), 64 slices × 3.75 mm and 3.27 mm interval, pitch 0.984:1, tube rotation 0.5 s. For D690, time-of-flight (TOF) and point spread function (PSF) were used as reconstruction algorithms; filter cutoff 5 mm, 18 subsets, three iterations. For D-STE, ordered subset expectation maximization (OSEM) was applied; filter cutoff 5 mm, 21 subsets, two iterations. Patients were instructed to void before imaging acquisition, and no oral or intravenous contrast agents were administrated or bowel preparations were used for any patient. The PET scans were analyzed visually and semi-quantitatively by a reader with experience (more than 10 years) in this field (DA) by measuring eight metabolic metrics: the maximum standardized uptake value corrected for body weight (SUVmax), mean SUV corrected for body weight (SUVmean), maximum standardized uptake value lean body mass (SUVlbm), maximum standardized uptake value body surface area (SUVbsa), lesion to liver SUVmax ratio (L-L SUV R), lesion to blood-pool SUVmax ratio (L-BP SUV R), metabolic tumor volume (MTV), and total lesion glycolysis (TLG) of the SPN. The workstation used for the measurements for SUV-related parameters was Xeleris 3.1 GE.

UVmax of the liver was calculated at the VIII hepatic segment of transaxial PET images using a round-shape 10 mm region of interest (ROI); SUVmax of the blood-pool was calculated at the aortic arch by use of transaxial PET images with a round-shape 10 mm ROI not involving the vessel wall. MTV was measured at the volume of interest (VOI) of SPN from attenuation-corrected ^18^F-FDG-PET images using a SUV-based automated contouring program (Advantage Workstation 4.6, GE HealthCare) with an isocounter threshold method based on 41% of the SUVmax, as previously recommended by the European Association of Nuclear Medicine because of its high inter-observer reproducibility [28]. Then, TLG was derived as the product of MTV and its SUVmean.

### 2.3. Texture Feature Extraction

Textural features were calculated using the LIFEx 2.20 package (http://www.lifexsoft.org 10 September 2021) [29] on PET images using the same procedure explained above, with similar VOI after a new segmentation process. A total of 42 RFs were extracted from the PET images (Table S1) divided in first-order statistics (histogram-related and shape-related) and second-order statistics (grey level co-occurrence matrix, GLCM related, grey-level run length matrix, GLRLM related, neightborhood grey level different matrix, NGLDM related, and grey-level zone length matrix, GLZLM related). LIFEx calculates RFs only for VOIs of at least 64 voxels. These measurements were performed by a reader (F.D.) with experience on this kind of analysis.

### 2.4. Statistical Analysis

**Figure 2:**
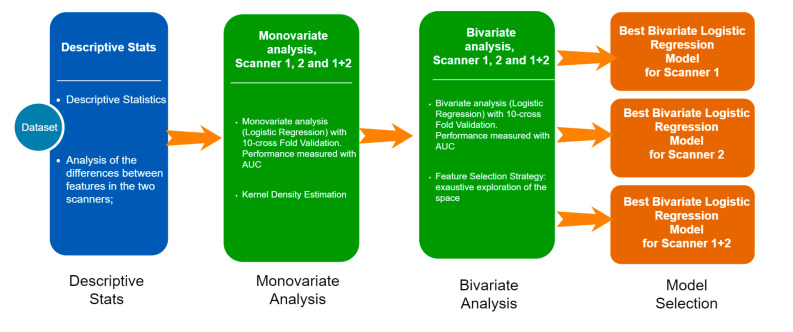
Résumé of statistical analyses performed.

Statistical analyses were performed out using MedCalc Software version 18.1 (8400 Ostend, Belgium) and R (http://www.R-project.org/). In the descriptive analysis, the categorical variables were represented as simple and relative frequencies, while the numeric variables as mean, standard deviation, and range values. For each scanner, the kernel density estimation built on the radiomics feature values were qualitatively compared, and the presence of significant differences were evaluated with the Wilcoxon–Mann–Whitney test. The general statistical pipeline in shown in Figure 2 and is composed of the following steps:

- Univariate analysis: a univariate analysis was performed on three different sets; the set of patients were treated with Scanner 1, Scanner 2, and the entire dataset. The aim of this step was to figure out how different technologies can affect the relationship between each RF and the clinical outcome.
- Bivariate analysis: with the aim of developing three predictive models (Scanner 1, Scanner 2, and for both the scanners), we analyzed the entire set of the possible couples of variables (the Cartesian product of the radiomics and the main clinical features, such as age, gender, nodule size, side). For each couple of variables, we calculated the bivariate logistic regression model and then we ranked them on the basis of the area under the curve (AUC) under the receiving operator curve (ROC) after a 10-cross fold validation training/testing test.
- Model selection: the best bivariate logistic regression model was selected for Scanner 1, Scanner 2, and Scanner 1 + 2 on the basis of the highest AUC.

AUC higher than 0.8 was arbitrarily considered optimal to predict the final diagnosis of SPN.

## 3. Results

### 3.1. Patients Characteristics

**Figure 3:**
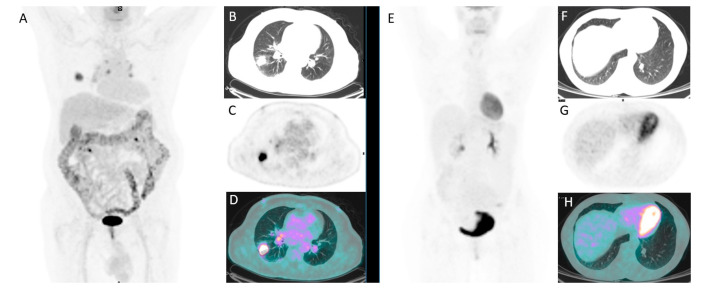
A 77-year-old male with a SPN of 22 mm of diameter in the right lung and increased FDG uptake (**A**–**D**). Another case of a 54-year-old female with a 12 mm lung nodule in the inferior lobe of the left lung without significant FDG uptake (**E**–**H**).

In total, 202 patients were included in the study (Table 1). Average age was 68 (range 37–86); there was a higher prevalence of males (*n* = 117). SPNs were more frequently in the right side and in the upper lobe. The mean diameter max was 20.6 mm (range 10–29 mm) and the mean volume 3861 mm^3^ (197–17,432 mm^3^). One-hundred and twelve (55%) studies were acquired on Discovery STE tomograph, while the remaining 90 (45%) were acquired on D690 tomograph. At the visual analysis, 140 (69%) PET/CT resulted positive, showing the presence of an increased radiotracer uptake higher than the background (surrounding lung tissue and blood pool activity) corresponding to the SPN (Figure 3). Of 140 positive PET/CT, 79 were acquired on scanner DSTE and 61 on scanner 690. The mean SUVmax, SUVmean, SUVlbm, SVUbsa, L-L SUV R, L-BP SUV R, MTV, and TLG were 7, 4.6, 5.3, 1.8, 2.5, 3.1, 5.5, and 22.2, respectively. The final diagnosis was malignant in 127 cases (63%), benign in 64 (32%), and indeterminate in the remaining 11 (5%). Among PET/CT studied performed on scanner DSTE, 70 (62.5%) had a final diagnosis of malignancy (48 adenocarcinoma, 10 squamous cell carcinoma, 12 other), 35 (31%) of benign disease, and 7 (6.5%) of indeterminate nature. Instead, for scanner 690, 57 (63%) had a diagnosis of malignancy (38 adenocarcinoma, 7 squamous cell carcinoma, 12 other), 29 (32%) of benignity, and 4 (5%) as indeterminate. No significant differences considering the final diagnosis, the oncological subtype, and PET/CT results between the two scanners were registered (*p =* 0.345, *p* = 0.444 and *p* = 0.765). Among malignant lesions, the most common histotype was adenocarcinoma (*n* = 86), followed by squamous cell carcinoma (*n* = 17) and neuroendocrine tumor (*n* = 12); rarer singular cases of large cell carcinoma and sarcomatoid carcinoma were reported. The remaining 10 lesions were classified as malignant after cytological examination (Table 2). Instead, of 64 benign lesions: 42 had a cytological negative examination and did not undergo surgery, and 7 had a final diagnosis of hamartoma, 6 of inflammation, 2 of granuloma, and 2 of solitary fibrous tumor (Table 2).

### 3.2. Comparison between the Two PET/CT Scanners

**Figure 4:**
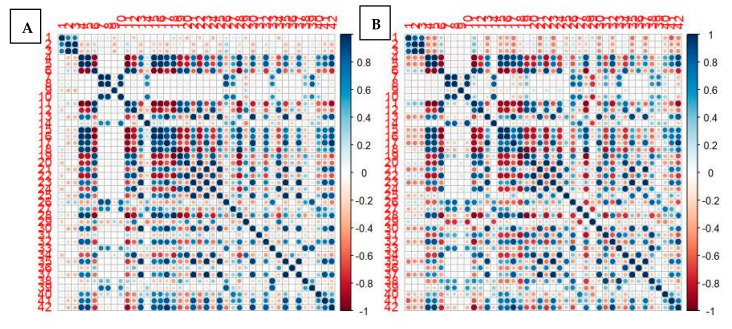
Correlation map for all first-order and second-order radiomics features between the two scanners (scanner DSTE (**A**); scanner 690 (**B**)). Red means high positive correlation; blue means high negative correlation; white means no correlation.

The main clinical and epidemiological characteristics (age, gender, SPN size) were not significantly different between the two PET/CT tomographs (D690 and D-STE) (Table 3). Instead, all PET/CT features (SUVmax, SUVmean, SUVlbm, SUVbsa, L-L SUV R, L-BP SUV R, MTV, and TLG) were significantly different; in particular, they were significantly higher in patients who performed scans on the 690 scanner. Among all RFs, 31/42 features were significantly different among the 690 and D-STE scanners. Only histo skewness, histo kurtosis, histo excess hurtosis, shape volume mL, shape volume vx, shape compacity, GLCM correlation, NGLDM coarseness, GLZLM SZLGE, GLZLM GLNU, and GLRLM RLNU were concordant between the two tomographs. However, the correlation map for the cross correlation between all radiomics features between the two scanners was quite similar (Figure 4).

### 3.3. Prediction Accuracy

At univariate analysis (Table 4), for scanner 1 (690), all PET metrics except of MTV had an optimal AUC to predict the final diagnosis of SPN; among radiomics first-order features, only Histo entropy_log 10, Histo entropy_log 2, and Histo energy had AUCs above 0.8. Instead, among radiomics second-order features, most of them were shown to have a strong impact in predicting malignancy (all except GLCM correlation, GLRLM RP, and GLZLM SZLGE). For scanner 2 (D-STE), all mean AUCs of parameters were lower than scanner 1, despite good performances, and only three grey-level zone length matrix parameters (GLZLM ZP, GLZLM GLNU, and GLZLM LZE) had an accuracy with AUC > 0.8. The combination of two scanners revealed that the features with the best accuracy were founded for PET parameters: L-BP SUV R, SUVbsa, SUVlbm, and L-L SUV R. After bivariate analysis (Table 5), for each scanner and scanners combined, the best combinations between all metabolic and radiomics features are described in Table 5. The accuracies for scanner 690 were higher compared to scanner D-STE and scanner 1 + 2 (Figure S3). For scanner 690 (Figure S1), a combination between GLCM-related features, histogram-related features, and GLZLM-related features presented the best performances. For scanner D-STE (Figure S2), a combination between GLCM-related features and histogram features were confirmed to have a fundamental impact in the prediction, together with MTV and other PET-related metrics. For scanner 1 + 2 (Figure S3), the best features were histogram-related features, SUV metrics, and MTV. However, combinations with other radiomics parameters also demonstrated good accuracy but were less significant.

## 4. Discussion

In this paper, we tested and compared the diagnostic accuracy of different PET and RF features with the aim of investigating their ability to distinguish between malignant and benign SPNs. Most of these metabolic and radiomics variables demonstrated optimal accuracy with AUC > 0.8, and also at the multivariate analysis, several combinations of them showed optimal performances (see Table 5).

SPN may be a diagnostic challenge due to the absence of noninvasive strong factors as predictors of the nature of these nodules, with the final diagnosis often occurring after biopsy [30]. In this field, potential instruments able to predict the nature of SPN without invasive procedures (such as biopsy) may be fundamental. Moreover, biopsy is the reference standard for the classification of a lesion, but it presents several limitations: it is a procedure potentially associated with complications, it is invasive, it cannot provide spatial information, it is usually not able to repeat, it can be not representative of the entire lesion because it captures a small portion the lesion, and it requires hospitalization with a consequent cost increase for healthcare systems.

On the other hand, RF gives the prospect of performing an analysis of the whole lesion in all parts in a noninvasive way [31]. The interpretation of medical images is directly related to the observer experience and expertise. The visual analysis, which was considered the gold standard for decades, for the diagnosis of several oncological diseases seems to be too limited and not free of errors [7]. Thus, the need for a more objective and accurate analysis of medical images had to be fulfilled in order to determine reliable imaging biomarkers, which led to the development of radiomics and its texture features.

In the setting of differentiation between malignant and benign SPN, several papers [19,20,21,22,23,24] showed a positive impact of PET RFs with many different features proposed as accurate. Often, more than one RF showed optimal diagnostic performance that was even better through applying a combination of RFs. These pieces of evidence are in agreement with our results, wherein we demonstrated that many of PET and RF features had a significant role in predicting the nature of SPNs. Among PET features, SUV-related parameters had better diagnostic performances than MTV and TLG. Instead, among RFs, almost all (first-order and second-order statistics) showed a high accuracy with rare exceptions. Features with AUC less than 0.6 were only histo kurtosis, histo excess kurtosis, and GLCM correlation parameters (Table 4). Probably one single radiomic parameter is not sufficient to properly describe the gross heterogeneity of a tumor since the gross texture consists of multiple patterns and characteristics. For this reason, a combination of different texture parameters (such as a radiomics signature) may better represent the SPN identity and guide the diagnosis. RFs on FDG provide different types of data that should be used together with more classical SUV-related metrics for making a diagnosis. These features combined might cause a significant improvement in discriminating benign from malignant SPN over commonly used clinical metrics and qualitative analysis.

Another point that emerged from our analysis is the influence of PET scanner in the measurement of RFs, an issue not well investigated in the literature [32,33]. In clinical practice, it is not so rare to have different PET scanners in the same department; for example, tomographs from different manufacturers or different models from the same manufacturer. These scanners may have significant structural differences related to the geometrical and components characteristics, but also could be related to the acquisitions and reconstruction protocols [34,35,36]. For example, the application of specific filters such as TOF or PSF may gain the detection of the signal, improving the accuracy. In our centers, we have two different PET scanners with similar structural features but different reconstruction filters, such as scintillator crystal, which affects their performances [36,37].

To avoid a possible difference in the type of patients studied between the two scanners, we compared the main features of these patients (PET results, final diagnosis, subtypes of tumor), finding no differences between the two groups. This evidence strengthens the impact of PET scanner of RF measurement.

However, despite these technical differences, the cross correlation of PET RFs between the two tomographs was very similar (Figure 1), and the RFs derived as most accurate in the prediction of final diagnosis were quite identical. GLCM- and histogram-related features were among the most significant for both scanners considered individually or jointly. For scanner 2, MTV also was shown to be very accurate, probably due to the fact that scanner 2 was more sensitive in signal detection and in the measurement of SUV and similar parameters. Compared with PET classical features (such as SUVmax, SUVmean), RFs did not show a huge predominance in distinguishing SPN nature, confirming the good performance of SUV-related variables in this field. Thus, it seems premature and excessive to suggest a routine use of RFs for SPNs; further studies including larger patient cohorts are warranted to confirm or controvert our results so that this noninvasive approach can be introduced into routine clinical practice. The increasing introduction into clinical practice of PET/CT tomographs with silicon photomultiplier (SiPM) technology will likely lead to new advances in the field of functional imaging radiomics, and studies are desirable in this direction. Our results underline the importance of the technology available for each institute and possible impact in the measurements of radiomics parameters. This aspect must be kept in mind when performing studies such as this. Thus, harmonizing the acquisition and reconstruction parameters between scanners and studies is a crucial step for future texture analysis.

Our study presents some limitations: first, the retrospective design of the study, which implies the use of PET/CT scanners that do not represent the current state-of-the-art models from a technological point of view; second, the relative low number of patients included, although it was comparable with that of previous studies; third, the heterogeneity of patients features included; and fourth, the use of a single software for RF analysis.

## 5. Conclusions

With this study, we have demonstrated that many different PET RFs were able to differentiate between malignant and benign SPN with high accuracy, but these parameters were directly dependent on the PET tomograph used and its features.

## Figures and Tables

**Table 1:** The main features of the patients included.

	*n* (%)
Age, mean ± SD (range)	68 ± 11 (37–86)
Male/female	117:85
Lung side right/left	128:74
Lung localization Upper lobe Inferior lobe Medium lobe Lingula	110 (54%) 68 (34%) 20 (10%) 4 (2%)
Diameter max mm, mean ± SD (range)	20.6 ± 6.5 (10–29)
Volume mm^3^, mean ± SD (range)	3861 ± 3578 (197–17,342)
Scanner PET Discovery 690 Discovery ST	90 (45%) 112 (55%)
PET/CT visual result Positive Negative	140 (69%) 62 (31%)
Mediastinal nodes FDG positive	60 (30%)
SUVmax, mean ± SD (range)	7 ± 7.3 (0,52–61.4)
SUVmean, mean ± SD (range)	4.6 ± 5.5 (0.32–52)
SUVlbm, mean ± SD (range)	5.3 ± 5.5 (0.34–45)
SUVbsa, mean ± SD (range)	1.8 ± 1,8 (0.13–14)
Lesion to liver SUV ratio, mean ± SD (range)	2.5 ± 2.5 (0.17–19.13)
Lesion to liver SUV ratio, mean ± SD (range)	3,1 ± 3.2 (0.22–23.1)
MTV, mean ± SD (range)	5.5 ± 5.2 (0.6–34,2)
TLG, mean ± SD (range)	22.2 ± 39.2 (0.32–432)
Final diagnosis Benign Malignant Indeterminate	64 (32%) 127 (63%) 11 (5%)

**Table 2:** Final histological/cytological diagnosis of SPNs.

	*n* (%)
MALIGNANT *n* = 127	
Adenocarcinoma	86 (68%)
Squamous cell carcinoma	17 (13%)
Large cell carcinoma	1 (1%)
Neuroendocrine tumor	12 (9%)
Sarcomatoid carcinoma	1 (1%)
Unspecified	10 (8%)
BENIGN *n =* 64	
Hamartoma	7 (11%)
Inflammation	6 (9%)
Solitary fibrous tumor	2 (3%)
Granuloma	2 (3%)
Hamatochondroma	1 (1.5%)
Angioma	1 (1.5%)
Lipoma	1 (1.5%)
Active tuberculosis	1 (1.5%)
Fibrosis	1 (1.5%)
Negative cytological examination	42 (66.5%)

**Table 3:** Comparison of the clinical, epidemiological, metabolic, and radiomics feature distributions between the two PET scanners.

SCANNER D690 vs. D-STE			
Clinical-Epidemiological Features	*p*-Value	Second-Order Statistics	*p*-Value
Age	0.659	GLCM entropy_log10	<0.001
Gender	0.659	GLCM entropy_log2	<0.001
Size	0.746	GLCM dissimilarity	<0.001
**“Conventional” PET features**		GLRLM SRE	<0.001
SUVmax	<0.001	GLRLM LRE	<0.001
SUVmean	<0.001	GLRLM LGRE	0.001
SUVlbm	<0.001	GLRLM HGRE	<0.001
SUVbsa	<0.001	GLRLM SRLGE	0.002
L-L SUV R	0.003	GLRLM SRHGE	<0.001
L-BP SUV R	0.009	GLRLM LRLGE	<0.001
MTV	<0.001	GLRLM LRHGE	0.006
TLG	<0.001	GLRLM GLNU	<0.001
**First-order statistics**		GLRLM RLNU	0.052
Histo skewness	0.316	GLRLM RP	<0.001
Histo kurtosis	0.758	NGLDM coarseness	0.799
Histo excess kurtosis	0.758	NGLDM contrast	<0.001
Histo entropy_log10	<0.001	NGLDM busyness	<0.001
Histo entropy_log2	<0.001	GLZLM SZE	<0.001
Histo energy	<0.001	GLZLM LZE	<0.001
Shape volume_mL	0.917	GLZLM LGZE	0.003
Shape volume_vx	0.917	GLZLM HGZE	<0.001
Shape sphericity	0.037	GLZLM SZLGE	0.877
Shape compacity	0.859	GLZLM SZHGE	<0.001
**Second-order statistics**		GLZLM LZLGE	<0.001
GLCM homogeneity	<0.001	GLZLM LZHGE	0.002
GLCM energy	<0.001	GLZLM GLNU	0.803
GLCM contrast	< 0.001	GLZLM ZLNU	<0.001
GLCM correlation	0.052	GLZLM ZP	< 0.001

**Table 4:** Univariate analysis for all PET and RFs and each scanner considered alone or in combination.

	Mean AUC		
	Scanner 1	Scanner 2	Scanner 1 + 2
**“Conventional” PET features**			
SUV-related			
SUVmax	0.855	0.714	0.760
SUVmean	0.840	0.714	0.752
SUVlbm	0.851	0.730	0.767
SUVbsa	0.859	0.724	0.769
L-L SUV R	0.847	0.727	0.766
L-BP SUV R	0.860	0.727	0.776
Metabolic volumes			
MTV	0.594	0.601	0.562
TLG	0.771	0.669	0.633
**First-order statistics**			
Histogram-related			
Histo skewness	0.682	0.585	0.629
Histo kurtosis	0.584	0.591	0.560
Histo excess kurtosis	0.584	0.592	0.560
Histo entropy_log10	0.869	0.718	0.763
Histo entropy_log2	0.869	0.719	0.762
Histo energy	0.852	0.704	0.743
Shape-related			
Shape volume_mL	0.656	0.626	0.542
Shape volume_vx	0.656	0.626	0.542
Shape sphericity	0.615	0.641	0.580
Shape Compacity	0.637	0.634	0.530
**Second-order statistics**			
GLCM-related			
GLCM homogeneity	0.846	0.692	0.733
GLCM energy	0.846	0.702	0.743
GLCM contrast	0.849	0.705	0.740
GLCM correlation	0.572	0.677	0.556
GLCM entropy_log10	0.853	0.732	0.747
GLCM entropy_log2	0.850	0.734	0.747
GLCM dissimilarity	0.845	0.702	0.739
GLRLM-related			
GLRLM SRE	0.843	0.692	0.731
GLRLM LRE	0.841	0.676	0.723
GLRLM LGRE	0.813	0.688	0.733
GLRLM HGRE	0.819	0.695	0.725
GLRLM SRLGE	0.799	0.687	0.729
GLRLM SRHGE	0.828	0.698	0.730
GLRLM LRLGE	0.831	0.692	0.741
GLRLM LRHGE	0.762	0.678	0.686
GLRLM GLNU	0.752	0.752	0.663
GLRLM RLNU	0.719	0.705	0.642
GLRLM RP	0.483	0.680	0.722
NGLDM-related			
NGLDM coarseness	0.643	0.783	0.590
NGLDM contrast	0.808	0.808	0.721
NGLDM busyness	0.783	0.643	0.700
GLZLM-related			
GLZLM SZE	0.777	0.677	0.703
GLZLM LZE	0.821	0.821	0.704
GLZLM LGZE	0.785	0.785	0.722
GLZLM HGZE	0.793	0.689	0.722
GLZLM SZLGE	0.614	0.614	0.574
GLZLM SZHGE	0.794	0.689	0.718
GLZLM LZLGE	0.837	0.697	0.725
GLZLM LZHGE	0.755	0.671	0.664
GLZLM GLNU	0.810	0.810	0.715
GLZLM ZLNU	0.808	0.716	0.726
GLZLM ZP	0.834	0.834	0.714

**Table 5:** Bivariate analysis regarding the prediction role of variables included.

Covariate 1	Covariate 2	Mean AUC
**Scanner 1**		
GLCM entropy_log10	GLZLM LZE	0.861
GLCM entropy_log2	GLZLM LZE	0.860
Histo entropy_log10	GLZLM LZE	0.858
GLCM homogeneity	GLZLM LZE	0.858
GLCM entropy_log2	GLZLM LGZE	0.856
GLCM entropy_log10	GLZLM LGZE	0.856
Histo entropy_log2	GLZLM LZE	0.856
GLCM entropy_log10	GLZLM LRE	0.854
Histo entropy_log2	GLZLM LGZE	0.852
Histo entropy_log10	GLZLM LGZE	0.852
**Scanner 2**		
Histo entropy_log10	Shape sphericity	0.734
Histo energy	L-BP SUV R	0.732
GLCM entropy_log2	MTV	0.728
GLCM entropy_log10	MTV	0.727
Histo energy	L-L SUV R	0.727
GLCM entropy_log10	GLRLM RLNU	0.727
GLCM entropy_log2	GLRLM RLNU	0.726
Histo entropy_log10	MTV	0.719
Histo entropy_log10	Histo entropy_log2	0.719
Histo entropy_log2	MTV	0.710
**Scanner 1 + 2**		
SUVmean	L-BP SUV R	0.785
SUVmax	L-BP SUV R	0.780
L-BP SUV R	MTV	0.774
Histo skewness	L-BP SUV R	0.771
SUVmean	SUVlbm	0.770
Histo energy	L-BP SUV R	0.769
SUVmean	SUVbsa	0.768
Histo entropy_log2	MTV	0.767
Histo entropy_log10	MTV	0.767
Histo energy	MTV	0.753

## Supplementary Materials

The following are available online at https://www.mdpi.com/article/10.3390/jcm10215064/s1, Figure S1: The best combination between RFs for scanner 1. Figure S2: The best combination between RFs for scanner 2. Figure S3: The best combination between RFs for scanner 1+2. Table S1: Summary of the radiomics features of PET/CT included in the study.
