# Supplementary material for: Role of 18F-FDG PET/CT Radiomics Features in the Differential Diagnosis of Solitary Pulmonary Nodules: Diagnostic Accuracy and Comparison between Two Different PET/CT Scanners
Source: J Clin Med. 2021 Oct 29;10(21):5064. doi: 10.3390/jcm10215064 (PMC8584460; doi:10.3390/jcm10215064)
Supplement: Supplementary file 1 [file jcm-10-05064-s001.zip › jcm-1390226-supplementary.pdf]

SUPPLEMENTARY TABLE S1: summary of the radiomics features of PET/CT included in the study

**“Conventional” PET features**
SUV related
SUVmax
SUVmean
SUVlbm
SUVbsa
L-L SUV R
L-BP SUV R
Metabolic volumes
MTV
TLG
**First-order statistics**
Histogram related
Histo skewness
Histo kurtosis
Histo excess kurtosis
Histo entropy_log10
Histo entropy_log2
Histo Energy
Shape related
Shape volume_mL
Shape volume_vx
Shape sphericity
Shape Compacity
**Second-order statistics**
Grey level co-occurrence matrix (GLCM) related
GLCM homogeneity
GLCM energy
GLCM contrast
GLCM correlation
GLCM entropy_log10
GLCM entropy_log2
GLCM dissimilarity
Grey-level run length matrix (GLRLM) related
GLRLM SRE
GLRLM LRE
GLRLM LGRE
GLRLM HGRE
GLRLM SRLGE
GLRLM SRHGE
GLRLM LRLGE
GLRLM LRHGE
GLRLM GLNU
GLRLM RLNU
GLRLM RP

Neighborhood grey level different matrix (NGLDM) related
NGLDM coarseness
NGLDM contrast
NGLDM busyness
Grey-level zone length matrix (GLZLM) related
GLZLM SZE
GLZLM LZE
GLZLM LGZE
GLZLM HGZE
GLZLM SZLGE
GLZLM SZHGE
GLZLM LZLGE
GLZLM LZHGE
GLZLM GLNU
GLZLM ZLNU
GLZLM ZP

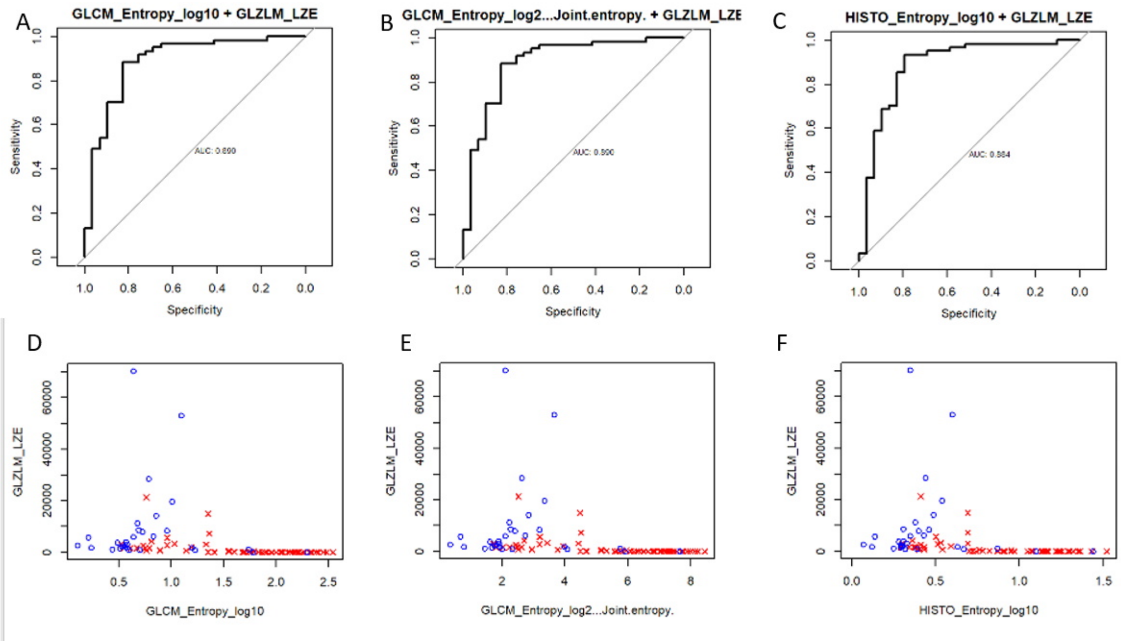

Supplementary Figure S1: the best combination between RFs for scanner 1

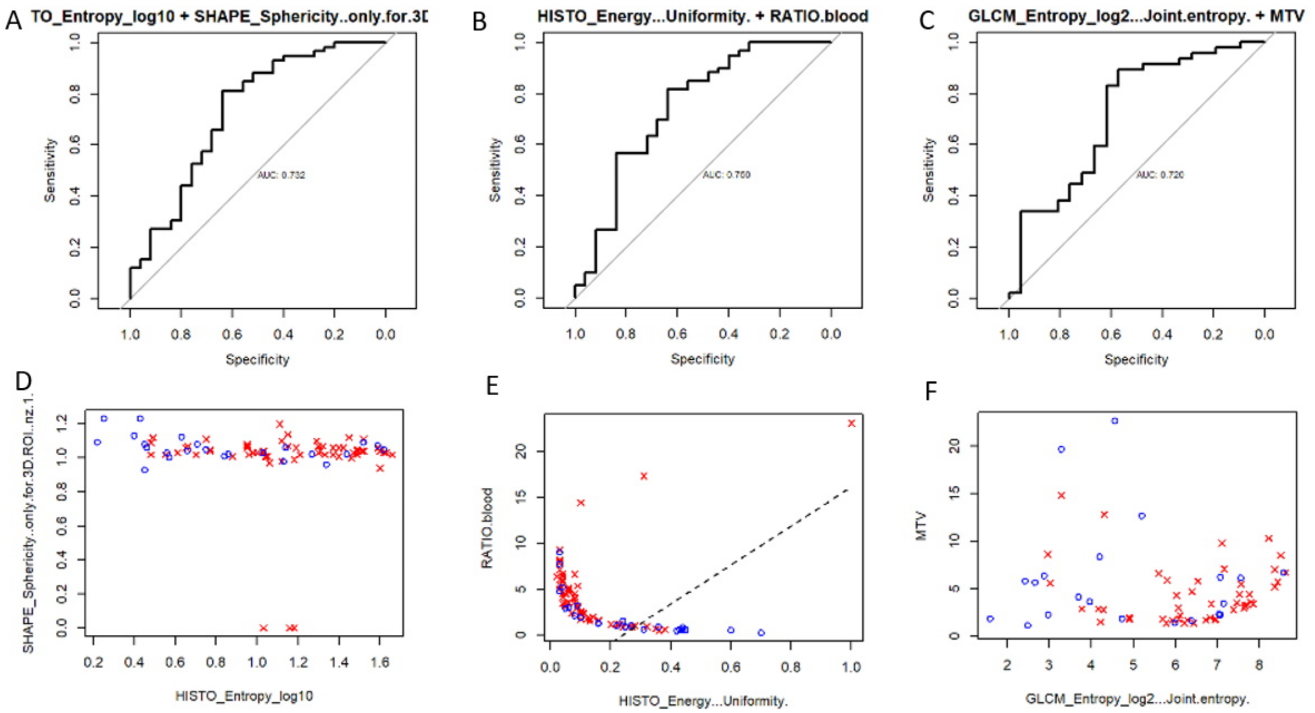

Supplementary Figure S2: the best combination between RFs for scanner 2

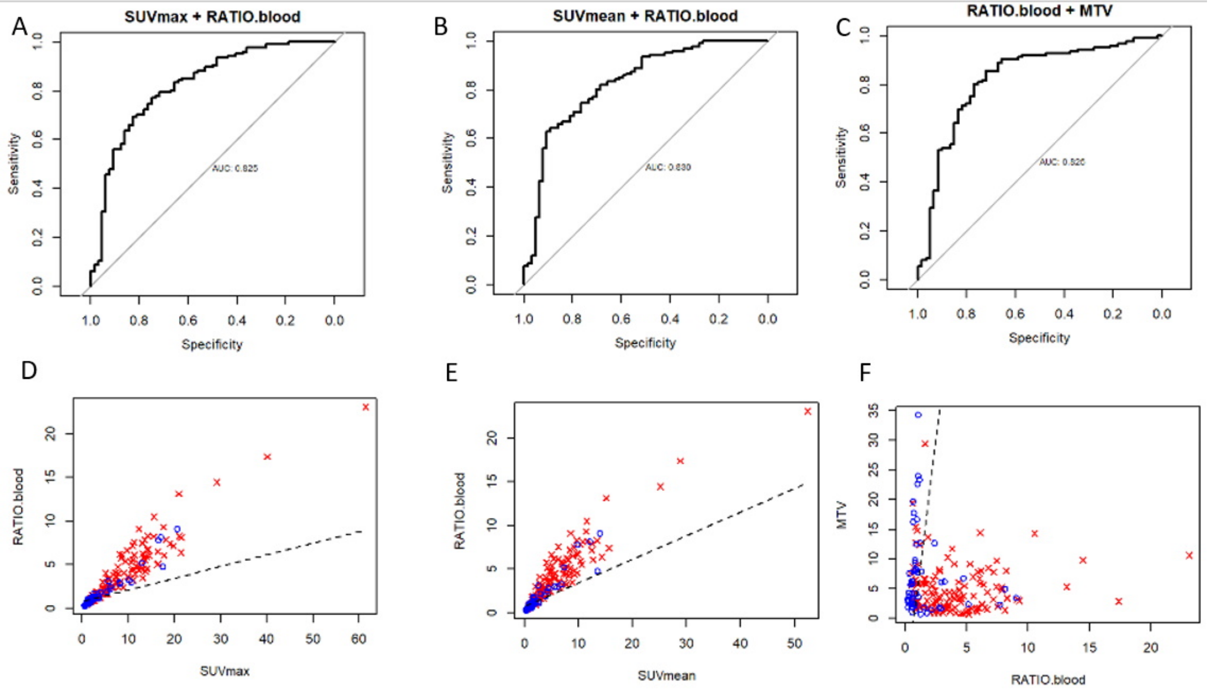

Supplementary Figure S3: the best combination between RFs for scanner 1+2
